# Yoga as an adjunct activity for medical students learning anatomy

**DOI:** 10.1186/s12909-022-03236-7

**Published:** 2022-03-17

**Authors:** Eugene C. Lee, William Adams, Noemy Sandoval-Skeet, Amy Hoyt, Kit Lee

**Affiliations:** 1 Department of Family Medicine, Loyola University Medical Center, 2160 South First Avenue, Maywood, IL 60153 USA; 2 MacNeal Family Medicine Residency, 3231 S. Euclid Ave. 5th Floor, Berwyn, IL 60402 USA; 3 Loyola University Chicago Stritch School of Medicine, 2160 South First Avenue, Maywood, IL 60153 USA; 4 Department of Medical Education, Loyola University Chicago Stritch School of Medicine, 2160 South First Avenue, Maywood, IL 60153 USA

**Keywords:** Yoga, Anatomy, Stress

## Abstract

**Background:**

Medical students experience high levels of stress during training due to demanding course loads which often leaves less time for self-care. This study combines the self-care technique of yoga with learning anatomical locations, innervations, actions, and functions of the muscles and organs to determine if anatomy tests scores are improved and whether students’ stress levels attenuate from participating in yoga.

**Methods:**

In this randomized controlled study, 64 student volunteers were randomized into either a yoga intervention group or wait list control group throughout the M1 anatomy course. The yoga group (*n* = 32) participated in 8 yoga sessions synced with the anatomy topics they were learning in lecture. The wait list group (*n* = 32) went through their normal anatomy curriculum but had an option to participate in the same yoga sessions after the anatomy course. The primary research purpose was to determine whether yoga improved anatomy exam performance by comparing four anatomy exam scores between the two groups. The secondary research purposes included the following: to determine whether yoga classes including anatomy teaching still conferred acute and long-lasting stress relief by, respectively, comparing a students’ own pre- and post-yoga stress level and self-perceived stress levels between the two groups; and to determine if a student’s confidence in anatomy was improved after a yoga session.

**Results:**

There was no significant difference in anatomy exam performance between students who received yoga and those on the waitlist (all *p* > 0.05). For students who received yoga, their average self-reported stress levels decreased after each yoga session, their average DASS (Depression, Anxiety and Stress Scale) score decreased after a yoga session, but they were not significantly less stressed than their waitlist peers prior to an exam, and their self-reported confidence in anatomy material related to the back, upper extremity, head and neck, and abdomen/pelvis increased.

**Conclusion:**

With this sample, there was no evidence that yoga sessions paired with anatomy lecture material improved overall anatomy exam performance, as opposed to only the musculoskeletal portion which other studies have looked at. However, yoga acutely reduced stress levels, and subjective feelings of knowledge improvement were noted by participants. Both of these can provide benefits to medical students.

## Background

Stress and anxiety are quite prevalent among medical students. One study found that among seven medical schools in The United States, over 80% of students experienced at least one form of distress which was defined as burnout, depressive stress, poor quality of life, and fatigue [1]. Some studies have found that almost 50% of medical students experience burnout [2, 3]. Specifically, during the COVID-19 pandemic in 2020, medical students reported a decline in their overall wellness [4], detriments to mental health [5], as well as worsened learning behaviors [5], forcing medical educators to consider effective and novel ways to train physicians [6].

Even before the COVID-19 pandemic though, many institutions have been creating wellness curricula to offer resources that improve students’ mental health. One survey found about 93% of medical school had a formal wellness program in 2019 [7], and suggestions for an effective wellness curriculum have been published [8, 9]. Interventions such as yoga and meditation for medical students have been previously found to improve feelings of peace, focus, and patience, while simultaneously reducing stress levels and fatigue [10, 11]. One study in particular evaluated a required one-time yoga/Pilates class within the first year anatomy course and found improvements comparing a pre- and post-test of upper and lower limb musculoskeletal information while still benefitting stress levels [12]. It is unknown if this enhanced musculoskeletal knowledge improved either students’ overall anatomy grade or standardized exams, which are a source of stress for medical students. Another study found that using yoga postures to teach lower limb musculoskeletal anatomy not only enhanced one’s anatomical knowledge, but facilitated the transfer of this new knowledge into one’s yoga practice [13]. However, it has been suggested that requiring wellness activities in addition to demanding academic curricula may have negative consequences, such as perceived loss of productivity, toxic positivity, and lack of effectiveness from medical students [14].

At the Stritch School of Medicine, yoga classes were offered to medical students prior to 2018, but attendance to the classes was low (averaging 2–5 students out of 150 +). When medical students were informally surveyed regarding the low attendance student comments generally focused on academic concerns. There were 4 main themes that emerged to increase likelihood of attending a yoga class: if the yoga class helped them study for class/exams (anatomy class was identified as the class most relevant to yoga by students) or prepared them for clinical rotations; if they felt yoga would fit their learning style; if they could get elective credit for attendance; and if the class fit their individual schedules. Of these themes, elective credit was not feasible, learning styles has been disproven to impact anatomy performance [15], and student wellness leaders had coordinated their own yoga classes that fit the students’ schedule better with minimally improved attendance. No research has yet to investigate whether teaching yoga to students could improve their overall anatomy exam performance (which may correlate better with standardized exams) while decreasing stress levels.

The primary purpose of the study was to determine if a Hatha yoga class synced with anatomy lectures (by including anatomical locations, innervations, actions, and functions of the muscles and organs from lecture) would enhance the process of learning anatomy reflected through test scores in the first year anatomy course of medical school. If true, this could help to address concerns identified from other studies [12, 14], such as participating in yoga is a loss of productivity or not effective for medical student stress and standardized tests. Positive results would hopefully increase student buy in for wellness programs and therefore improve participation. Because a medical school anatomy exam includes more information than just the material covered by the yoga class, two secondary purposes were included in the study. One secondary purpose was to determine if participating in yoga with embedded educational material would still confer the stress relieving benefits that other studies have identified. The other secondary purpose was to determine if students subjectively felt that their confidence in the anatomy material covered were improved.

## Methods

### Participants

During 2018–2020 academic years at the Stritch School of Medicine, all first year medical students were required to take anatomy in the first semester of medical school concurrently with two longitudinal courses. The anatomy course per week was run as follows: an average of 8 h of in-person lecture material with two or three in-person 4-h labs where students would rotate through and explore an already dissected body. This averages about 16–20 h of scheduled class time. About every three weeks, there was an anatomy exam regarding the past three weeks’ material. There were a total of four exams for the course.

The study included first year medical students at the Stritch School of Medicine (SSOM) who were enrolled in the fall semester anatomy course. The study excluded students with a medical condition or physical limitation that would preclude their ability to participate in yoga. All students enrolled in the anatomy course (335 over the 2 years) were notified via email of the study about one month prior to the start of the anatomy course. One week prior to the start of the anatomy course, interested students were invited to a question-and-answer session regarding the study. The research study was approved by the Loyola University Chicago Health Sciences Campus (LUCHSC) Institutional Review Board (IRB), and the research protocol was performed in accordance with the relevant guidelines and regulations of the LUCHSC IRB. All participation was voluntary, and all participants provided informed consent before participating in any study activities.

### Study design

All participants completed an intake survey to capture baseline characteristics, including their age, race, learning style, prior anatomy experience, and prior experience with yoga. For this study, traditional students were those who immediately matriculated to medical school while non-traditional students were those with a gap of at least one year between graduation and matriculation to medical school. Students’ primary learning style was also categorized as visual, aural, read/write, or kinesthetic as described by Fleming [16] and Leite et al. [17].

Before each anatomy exam, all participants also completed the Perceived Stress Survey 4 (PSS-4) which measures the frequency of stress on a scale from 0 to 16 points (where higher scores indicate more frequent stress) [18, 19]. Students assigned to the yoga intervention cohort completed eight yoga sessions. Before and after each session, these students self-reported their confidence in the anatomy content covered in that session ranging from 0 (no confidence) to 4 (very confident), stress on an 11-point score scale ranging 0 (no stress) to 10 (excessive stress), and completed the stress subscale of the Depression, Anxiety and Stress Scale (DASS-21) assessment; this subscale ranges from 0 to 42 points (where higher scores indicate more distress) [20].

Regarding the yoga sessions, a certified 200-h yoga teacher trained in Alignment yoga (a form of Hatha yoga) taught each yoga session. The teacher created a script that was utilized to maintain consistency between years. These sessions included both beginner and continuing yoga poses. The flow of the yoga followed the general sequence taught in Alignment yoga: Pre-yoga, standing postures, inversions, backbends, forward bends, twists, pranayama, and relaxation. Each yoga session included the anatomy lecture topics for that week. For example, if students had lectures on upper extremities, the yoga poses were described using anatomical locations, innervations, actions, and functions of the muscles of the upper extremity.

### Power and sample size calculation

An *a-priori* power and sample-size calculation was estimated to test the null hypothesis that average performance on the anatomy and physiology examinations was the same between students who take the yoga instruction course (intervention) and those who wait (control). Group sample sizes of 27 randomly assigned to the yoga condition and 27 randomly assigned to the control condition achieved 81.2% power to detect a difference of 7 (out of 100) examination points in a design with four examinations (or repeated observations). This computation assumed a 20% attrition rate in each group, a compound symmetry covariance structure for students’ correlated exam responses, and a standard deviation of 8.65 points. These assumptions were based on historical data from the course between 2016–2018. This computation also assumed the correlation between observations on the same subject was 0.80 and that the alpha level was 0.05 [21–23].

### Statistical analysis

First year medical students were randomized to the yoga or wait cohort using a 1:1 random allocation scheme. For these participants, demographics are provided as valid counts and percentages for all nominal characteristics and as mean with standard deviation for age. For the primary purpose a linear mixed-effects model was used to estimate the mean difference in exam performance between the experimental (Yoga) and control (Wait) groups. An interaction term was used to estimate the mean difference between these two cohorts for each exam, and a Sidak correction was used to control the Type 1 error rate. Because students could contribute up to four exam scores to the analysis, random intercepts were allowed for each student to account for their correlated exam performance. The degrees of freedom for these comparisons were estimated using the method of Kenward and Roger [24]. The same approach was used to compare group responses on the PSS-4 assessment.

Among students assigned to the yoga cohort, linear mixed-effects models were also used to estimate the mean change in both the self-reported stress response and DASS stress response from pre-intervention to post-intervention. As before, a Sidak correction was used to control the Type 1 error rate for these comparisons and, because students could contribute up to two responses for each yoga session, random intercepts were allowed for each student to account for their correlated responses. The degrees of freedom for these comparisons were also estimated using the method of Kenward and Roger [24].

Finally, we used generalized estimating equations (GEE models) to estimate the odds of reporting higher confidence in each anatomy construct post-session versus pre-session for students assigned to the yoga intervention cohort. These models specified a multinomial distribution with cumulative logit link for the ordinal confidence response and used robust standard errors to account for students’ paired (correlated) survey responses. As before, a Sidak correction was used to control the Type 1 error rate for these comparisons. All analyses were completed using SAS version 9.4 (Cary, NC).

## Results

Out of all 335 students eligible to participate in the study, 64 of the eligible students (19%) volunteered. This exceeded enrollment expectations with 32 (50%) participants assigned to the control (Wait) condition and 32 (50%) students assigned to the intervention (Yoga) condition. The average age was 24.6 (SD = 2.1) years. Most students identified as female (84%) and half identified as White (50%). Nearly all students were non-traditional students (84%), and most previously studied anatomy (64%). The majority of participants identified their learning style as multimodal (77%). Most students descibed their learning style as kinesthetic (77%), reading-oriented (59%), or visual-oriented (50%). Few participants identified their learning style as auditory (28%). Finally, the majority of students reported prior yoga experience (78%), which on sensitivity analysis did not affect exam performance between the two groups (*p* = 0.28). See Table 1.

**Table 1:** Participant characteristics

	Group		
	Wait (*n* = 32)	Yoga (*n* = 32)	Total (*N* = 64)
Mean age (SD)	24.3 (1.7)	24.9 (2.5)	24.6 (2.1)
Sex			
Female	27 (84.4%)	27 (84.4%)	54 (84.4%)
Male	5 (15.6%)	5 (15.6%)	10 (15.6%)
Race			
White	16 (50%)	16 (50%)	32 (50%)
Black	2 (6.3%)	5 (15.6%)	7 (10.9%)
Asian	12 (37.5%)	7 (21.9%)	19 (29.7%)
Hispanic	1 (3.1%)	0 (0%)	1 (1.6%)
Middle Eastern	0 (0%)	2 (6.3%)	2 (3.1%)
Multiracial	1 (3.1%)	2 (6.3%)	3 (4.7%)
Traditional Medical Student			
No	27 (84.4%)	26 (81.3%)	53 (82.8%)
Yes	5 (15.6%)	6 (18.8%)	11 (17.2%)
Prior Anatomy Experience			
No	10 (31.3%)	13 (40.6%)	23 (35.9%)
Yes	22 (68.8%)	19 (59.4%)	41 (64.1%)
Learning Style			
Visual	17 (53.1%)	15 (46.9%)	32 (50%)
Auditory	10 (31.3%)	8 (25%)	18 (28.1%)
Reading	19 (59.4%)	19 (59.4%)	38 (59.4%)
Kinesthetic	23 (71.9%)	26 (81.3%)	49 (76.6%)
Multimodal	26 (81.3%)	23 (71.9%)	49 (76.6%)
Yoga level			
New	6 (18.8%)	8 (25%)	14 (21.9%)
Beginner	8 (25%)	6 (18.8%)	14 (21.9%)
Continuing	6 (18.8%)	10 (31.3%)	16 (25%)
Intermediate	9 (28.1%)	7 (21.9%)	16 (25%)
Expert	3 (9.4%)	1 (3.1%)	4 (6.3%)

**Fig. 1:**
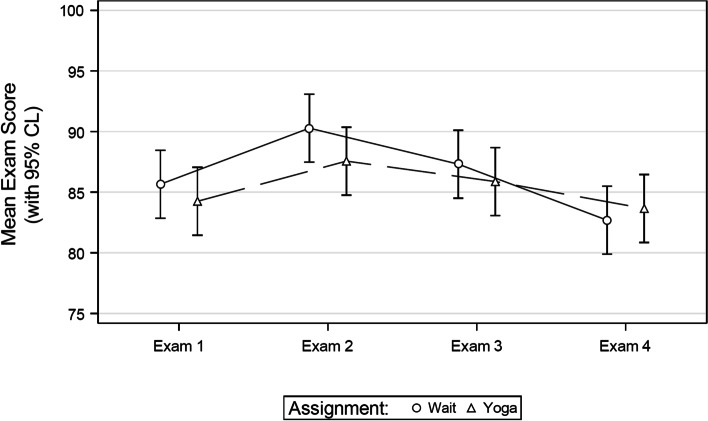
Mean exam scores by group assignment

**Fig. 2:**
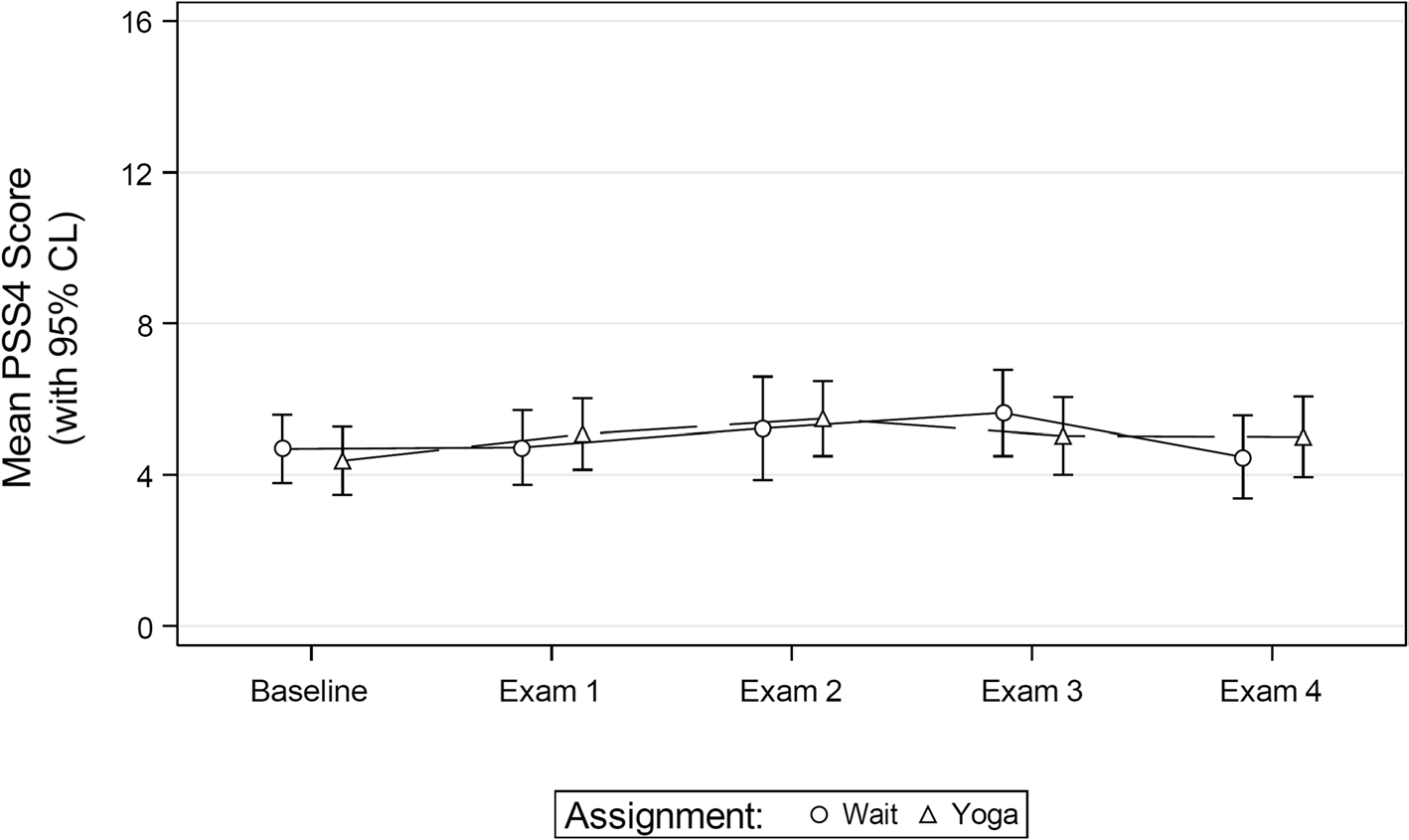
Mean PSS-4 scores before each exam by group assignment

With this sample, there were no significant differences in exam performance between the experimental (overall *M* = 85.34, SE = 1.21) and control (overall *M* = 86.48, SE = 1.21) groups for any of the four examinations (all *p* > 0.05) (Fig. 1). Additionally, there was no significant difference in the PSS-4 perceived stress score between the experimental (overall *M* = 4.97, SE = 0.39) and control (overall *M* = 4.99, SE = 0.40) groups prior to any of the four examinations (all *p* > 0.05)(Fig. 2).

However, among students assigned to the yoga cohort, self-reported stress levels significantly declined after each yoga session (all *p* < 0.05) and most DASS stress responses also declined (Table 2). Among students in the yoga cohort, confidence in anatomy knowledge was significantly higher after sessions focusing on the back (*OR* = 4.20; *p* = 0.002), upper extremity (*OR* = 2.66; *p* = 0.03), head and neck (*OR* = 18.38; *p* = 0.002), and abdomen and pelvis (*OR* = 2.49; *p* = 0.049) (Table 3).

**Table 2:** Self-reported stress and DASS scores among students assigned to the yoga intervention cohort

	**Valid S**	**Pre-Session (SD)**	**Post-Session (SD)**	**Mean Difference** **(95% CI)**	***P***
**Stress**					
Session 1	58	4.34 (2.16)	2.59 (1.66)	-1.76 (-2.61 to -0.91)	.002
Session 2	57	4.79 (2.27)	3.11 (2.11)	-1.53 (-2.32 to -0.73)	.002
Session 3	52	5.11 (2.03)	3.12 (1.67)	-1.94 (-2.84 to -1.04)	.002
Session 4	45	5.43 (2.31)	3.63 (2.34)	-2.05 (-3.16 to -0.94)	.002
Session 5	49	3.00 (2.06)	1.78 (1.28)	-0.88 (-1.61 to -0.15)	.01
Session 6	46	3.87 (2.36)	2.57 (2.00)	-1.15 (-1.85 to -0.45)	.002
Session 7	40	4.19 (2.69)	2.63 (2.34)	-1.53 (-2.49 to -0.57)	.002
Session 8	37	4.84 (2.73)	3.11 (2.32)	-1.77 (-3.12 to -0.42)	.01
**DASS**					
Session 1	58	13.38 (6.35)	8.00 (5.63)	-5.38 (-8.64 to -2.12)	.002
Session 2	57	14.00 (8.77)	7.79 (8.39)	-5.86 (-9.01 to -2.70)	.002
Session 3	52	12.74 (7.73)	6.92 (7.08)	-6.02 (-10.05 to -2.00)	.002
Session 4	45	15.91 (10.33)	9.45 (9.92)	-6.65 (-12.51 to -0.78)	.02
Session 5	49	9.00 (8.91)	3.48 (4.01)	-3.72 (-6.65 to -0.78)	.01
Session 6	47	13.25 (10.90)	6.78 (7.50)	-6.51 (-11.59 to -1.43)	.01
Session 7	40	14.10 (12.94)	9.37 (10.96)	-4.55 (-9.43 to 0.33)	.08
Session 8	37	18.63 (13.53)	11.22 (11.90)	-6.96 (-15.24 to 1.32)	.16

Valid S = Number of surveys used for the analysis. Confidence limits and significance values are adjusted using a Sidak correction. Sessions 1 = Back; 2 = Upper Extremity; 3 = Head and Neck; 4 = Thorax; 5 = Abdomen and Pelvis; 6 = Lower Extremity; 7 = Ascending and Descending Pathways; 8 = Cerebellum and Vestibular System

**Table 3:** Odds of higher confidence post-intervention versus pre-intervention for the yoga group

	**Valid S**	**Odds Ratio** **(95% CI)**	***P***
Session 1	58	4.20 (1.45 – 12.10)	.002
Session 2	57	2.66 (1.06 – 6.73)	.03
Session 3	52	18.38 (3.07 – 110.00)	.002
Session 4	45	1.84 (0.86 – 3.97)	.31
Session 5	49	2.49 (1.002 – 6.20)	.049
Session 6	47	2.17 (0.91 – 5.19)	.14
Session 7	40	2.48 (0.99 – 6.13)	.052
Session 8	37	2.66 (0.70 – 10.12)	.45

S = Number of surveys used for the analysis. Confidence limits and significance values are adjusted using a Sidak correction. Sessions: 1 = Back; 2 = Upper Extremity; 3 = Head and Neck; 4 = Thorax; 5 = Abdomen and Pelvis; 6 = Lower Extremity; 7 = Ascending and Descending Pathways; 8 = Cerebellum and Vestibular System

## Discussion

In 2014, the Association of American Medical Colleges (AAMC) found that medical students identifying higher levels of stress were more likely to be female, under-represented in medicine, Asian, sexual minorities, and those identifying as first generation in college [25]. Significantly more females participated in this study, which may be a reflection for those higher stress levels. The other identifiers are limited in this study either by the demographic make-up of the student body or not being asked in the study demographics. Additionally, this study enrolled more non-traditional students and those whose learning styles were multimodal. Although learning styles have been determined to not be correlated with anatomy performance [15], this identifier was included in the demographics because medical students still believe that learning styles impact academic performance. This is reflected by the disproportionate number of study volunteers identifying with kinesthetic learning. Although, not a purpose of this study, medical educators may want to consider what additional resources these groups may need to help them succeed in their education and help diversify the physician work-force. This may include helping medical students recognize that they should not focus on learning style, but rather specific strategies to learn material.

Similar to other studies [26–28], this study also found yoga lowers stress levels in health professionals (i.e., medical students) acutely. However, the stress reduction did not appear to be long-term as stress levels the day before the exam were comparable between the two groups. It is difficult to discern whether this is due to the addition of a learning component to the yoga sessions, the fact that sessions were not weekly in order to sync with the anatomy lectures, or because the amount of stress before an exam (i.e., when the comparison surveys between wait and control were administered) were similar between both groups regardless of intervention. In the future, a survey to compare stress levels at a neutral time may be beneficial to further elucidate this issue. This means that if yoga were to be used as a learning tool the stress benefits would not be lost.

The objective measure of tests scores were not statistically significant between the two groups. This is not surprising as the anatomy exams include non-yoga anatomy concepts, so many questions would not have been reviewed in the yoga session. Sensitivity analyses revealed that the two groups were comparable on individual MSK related test questions. This may account for the difference in findings from McCulloch, et. al [12] as they created a pre- and post-test regarding the material they taught in their yoga/pilates session. This study is more reflective of standardized testing that is more common in medical training. In addressing student concerns regarding using yoga class to perform better on exams, this study finds yoga class to not be effective for increasing exam scores compared to normal anatomy study material, but particpants in the experimental group did not score lower than in the control group, meaning that yoga class did not detract from study time. The combination of yoga (with or without an educational component) decreasing stress and not detracting from study time means that yoga should not be dropped from consideration as effective wellness curriculum continues to be explored.

The 7% difference in exams is a large difference. The study was created with two participant targets in mind. Because of randomization, 54 partcipants was the minimum number for study feasibility and statisical significance, but required exams between the two groups to vary by 7%. For a 5% difference in exams, 100 participants was needed. The study was on track to achieve the 100 participant target until the COVID-19 pandemic and subsequent changes to the Stritch curriculum (which included converting from two years of preclinical studies to one and a half years of preclinical studies). As these changes dramatically alter the number of yoga sessions and study consistency, it was determined that the minimum target was reached and to conclude participant enrollment.

Subjective measures indicate that students felt more confident in their anatomy knowledge by attending the yoga sessions, especially in the early sessions (sessions 1–3 and 5). This is consistent with findings from other studies using yoga to teach anatomy [12, 13]. Future studies may consider looking into if this translates to other areas of medical performance that are not specifically related to exams, such a confidence with performing a musculoskeletal exam, diagnosing musculoskeletal issues, or recommending musculoskeletal exercises to patients, especially as these topics would have more direct patient care impact as opposed to an exam and was identified by students as potentially being a motivator for attendance.

There was a low participation percentage in the study. Only 19% of eligible students enrolled. In practice, this replicates real life as not all students are interested in participating in wellness curriculums [12, 14]. Additionally, despite offering the same yoga sessions in the spring semester to the wait group, of the 32 participants assigned to wait for yoga instruction group only one student attended the spring sessions. Possible reasons for non-attendance include competing demands and low availability, feelings of needing to spend more time studying for other courses, and feeling like more personal time was needed. This highlights the fact that medical students desire a direct effect on their course/exam performance from activities that are adjuncts or supplemental to their routine curriculum.

Limitations of the study include the fact that the valid S (number of surveys used) decreased as the sessions progressed. This is due to students in the experimental group not attending sessions (either due to illness or forgetting about the session), attending the session but forgetting to complete the survey(s), and not attending sessions after a certain time due to conflicting demands/changes in personal schedules. Missing one session accounts for almost 50% of covered yoga material for the corresponding exam. With respect to surveys, those in the wait group were more likely to miss a survey. This shows that maintaining engagement with the wait group was difficult. In an attempt to address this, we used mixed-effects models that capitalize on all available data (rather than data solely from students who completed all study activities).

## Conclusion

Medical students, especially those identifying as kinesthetic learners, are yearning for teaching styles other than traditional lectures. These students need to be informed that more high yield resources exist that are not connected to learning style [15]. Looking at the bigger picture of medical education, additional attempts to help medical students recognize that although 1 h / week of yoga may not directly improve anatomy course test scores, there are still multiple benefits that they can gain from participating. These benefits include decreasing stress levels (both by self-reported levels and measured objectively via a modified DASS21) and improved subjective feelings of knowledge and comfort with anatomy material. This attempt should be extended to other wellness curriculum where students feel loss of productivity, poor buy-in, or lack of direct impact to testing scenarios.

Considerations for the future may be comparing an in-person yoga session focusing on anatomy terminology with that of a pre-recorded yoga session that students can repeat and review the anatomy related to yoga at their own pace. Additionally, medical schools will need to invest more than a few sessions of yoga to the anatomy topic to see a change in anatomy test scores.

## Data Availability

The datasets generated during and/or analyzed during the current study are available from the corresponding author on reasonable request as institutional policy dictates that a data use agreement with Loyola will need to be signed to share the data.

## Abbreviations

DASS: Depression, Anxiety and Stress Scale
PSS-4: Perceived Stress Survey 4
